# Determining Biventricular Repair Feasibility in Children with Dominant Right Ventricle Using Left Ventricular Quality Measured on Cardiac Computed Tomography

**DOI:** 10.31083/j.rcm2403092

**Published:** 2023-03-16

**Authors:** Monal Yu-Hsuan Chang, Jou-Hsuan Huang, Wen-Jeng Lee, Shu-Chien Huang, Yih-Sharng Chen, Jou-Kou Wang, Shyh-Jye Chen

**Affiliations:** ^1^Department of Medical Imaging, National Taiwan University Hospital, 10002 Taipei, Taiwan; ^2^Department of Surgery, National Taiwan University Hospital, 10002 Taipei, Taiwan; ^3^Department of Pediatrics, National Taiwan University Hospital, 10002 Taipei, Taiwan

**Keywords:** congenital heart disease, computed tomography, dominant right ventricle, biventricular repair

## Abstract

**Background:**

Left-ventricular (LV) characteristic measurements are crucial for evaluating the feasibility of biventricular repair (BiVR). This study aimed to determine the threshold of LV quality on cardiac computed tomography (CCT) for BiVR in children with a dominant right ventricle (DRV).

**Methods:**

We retrospectively reviewed all children with a DRV who underwent either BiVR or single ventricle palliation (SVP) at our institution between 2003 and 2019 in a case-control study with healthy individuals. Measurements including LV end-diastolic volume (LVEDV, mL), LV myocardial mass (LVMM, gm), and mitral annulus area (MAA, ${\mathrm{cm}}^{2}$) were quantified using CCT. The factor with the highest correlation with body size was used to adjust these three measurements to derive normal references in the control group. The LV quality of patients on each CCT measurement was represented as a percentage of the normal reference data that we established. The feasible LV quality for BiVR was defined as the lowest limit of all three LV measurements in one subject who survived BiVR among our patients with DRVs.

**Results:**

The cohort comprised 30 patients and 76 healthy controls. Height was the factor with the highest correlation with all three LV measurements. Height-adjusted normal reference curves and formulas were created. The mean LV quality in surviving patients who underwent BiVR was better than that in those who underwent SVP. The lowest limits for LV quality in one survivor of BiVR were 39.1% LVEDV, 49.0% LVMM, and 44.9% MAA. During follow up, the LV quality of patients who received BiVR shifted to the normal range.

**Conclusions:**

LV quality should be at least greater than 45% of normal values to promise survival in patients with DRVs who are being considered for a BiVR.

## 1. Introduction

Biventricular repair (BiVR) is hemodynamically more efficient than single ventricle palliation (SVP) (i.e., Fontan procedure and bidirectional Glenn procedure). However, selecting between BiVR and SVP may be difficult in ambiguous cases with small left ventricles (LV) and remains one of the most persistent challenges faced by pediatric cardiac surgeons [1, 2]. Numerous studies have introduced parameters to represent LV quality that may guide this decision in patients with a dominant right ventricle (DRV) [3, 4, 5, 6, 7, 8, 9, 10]. However, the predictors of LV quality to ensure successful BiVR with patient survival are not well defined [11]. Therefore, SVP is considered the safer option. However, the long-term outcomes after Fontan reconstruction are unsatisfactory [12].

Cardiac computed tomography (CCT) has been demonstrated as a powerful tool for structural analysis in congenital heart disease (CHD) [13, 14, 15]. Studies have reported that quantification of ventricular characteristics using CCT in adults is less invasive and has higher accuracy than cardiac catheterization [16]. The determination of functional and anatomical characteristics of the LV using CCT in children remains relatively unexplored [17, 18]. We propose that measurements of LV characteristics are crucial for selecting BiVR as a surgical option, particularly in patients with DRVs [19, 20]. These measurements are LV end-diastolic volume (LVEDV, mL), LV myocardial mass (LVMM, gm), and mitral annulus area (MAA, ${\mathrm{cm}}^{2}$), which represent the LV blood volume capacity, power of LV muscle, and patency of LV inflow, respectively. This study aimed to determine the threshold of LV quality measured using CCT for successful BiVR in children with a DRV.

## 2. Material and Methods

### 2.1 Participants

National Taiwan University Hospital Research Ethics Committee approved this retrospective study and waived the need for informed consent. This study was conducted at a single tertiary center with analysis of CCT images from July 2003 to January 2017 and clinical follow up until January 2019. The study was divided into three steps. Step I was to explore a simple corrective factor to adjust CCT measurements in children of various sizes. Based on the results from Step I, Step II involved building reference curves and a formula for normal LV quality on the proposed CCT measurements. Finally, Step III involved demonstrating the characteristics of LV quality (represented as a percentage to normal references) and tracing the outcomes using CCT measurements in patients with DRVs who received either BiVR or SVP.

Control subjects with a “normal” heart were included in the Step I and Step II control groups, which were used to establish normal references. The indications for CCT studies in the control group included airway problems, mediastinal lesions, or abnormal shadows on echocardiograms, which were eventually determined as ‘normal’ using CCT, or that were due to other minor confirmed pathologies on CCT images that did not affect the heart itself, such as small valvular vegetations. For the Step III group, patients with CHD with a DRV who underwent cardiac CCT were included in the preoperative and postoperative stages.

### 2.2 CCT Examinations, Post-Processing Techniques, and Quantification

Three CCT scanner models were used for this study (LightSpeed 16: GE Medical Systems, Milwaukee, WI, USA, Jul 2003–Jun 2006; LightSpeed 64 VCT: GE Healthcare, Waukesha, WI, USA, Jul 2006–Oct 2008; Sensation 64: Siemens Medical Solutions, Forchheim, Germany, Nov 2008–Jan 2017). Scanning was performed using electrocardiography triggering to inhibit cardiac-related motion artifacts. Slice thickness ranged from 0.625–0.8 mm. The matrix size in the X–Y plane was 512 × 512 pixels. The X-ray tube current was adjusted according to patient body weight to follow the principle of “as low as reasonably achievable” [13, 21]. The mean effective radiation doses range from 2.0 mSv in newborns to 15.6 mSv in young adults [21]. A nonionic iodinated contrast medium was prescribed according to patient body weight. Chloral hydrate was administered to uncooperative patients (aged <5 years old).

**Fig. 1.**
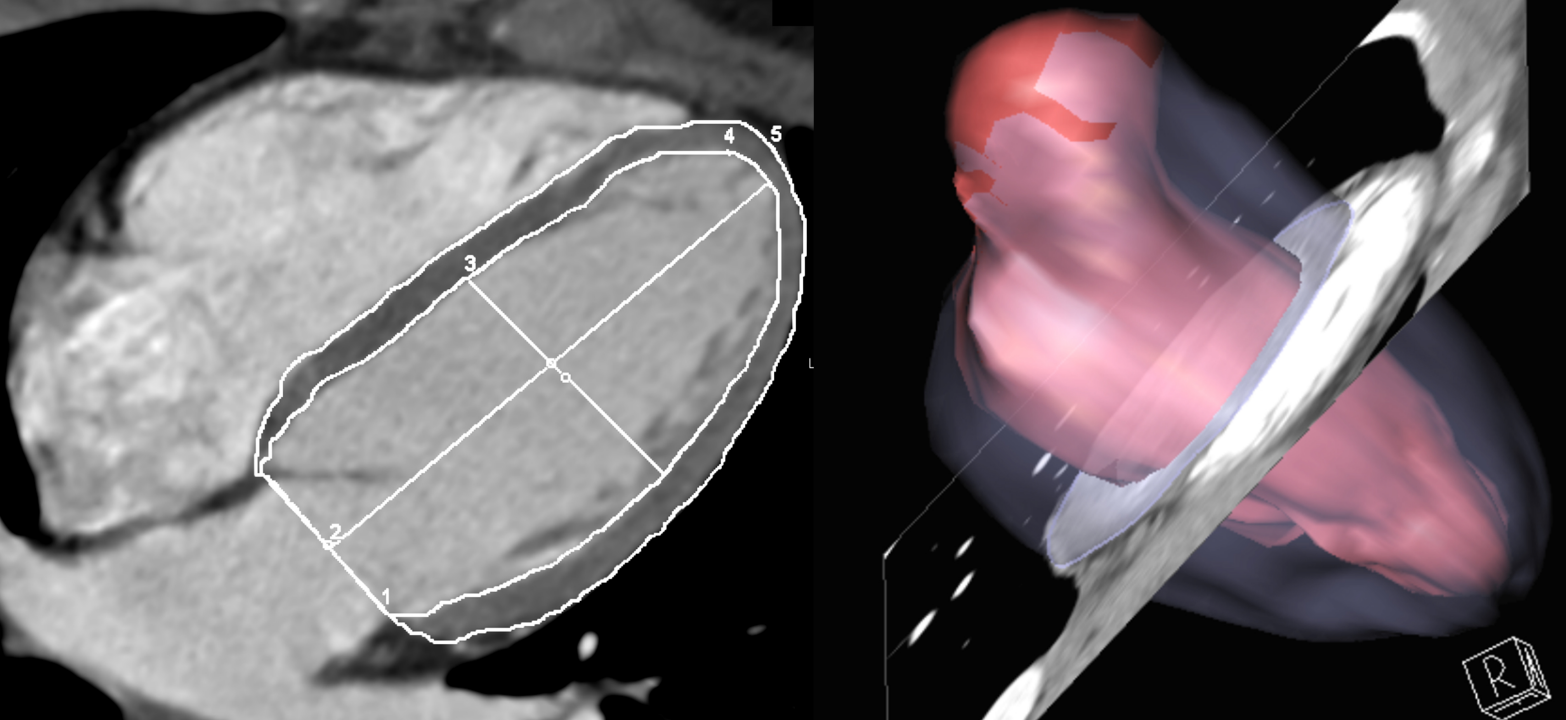
**Left ventricular quality measured at end-diastole cardiac computed tomography images**.

Post-processing data quantification was performed using a commercial software (Syngo®; Siemens Medical Solutions, Forchheim, Germany). Three-dimensional volume-rendering images were used to quantify the LVEDV and LVMM. The MAA was measured in two dimensions. All measurements were performed during end-diastole (Fig. 1). A single reader with 23 years of experience in pediatric CCT interpretation assessed the CCT images and obtained the required measurements [22, 23, 24]. The reader was unaware of the participant outcomes while obtaining the measurements.

### 2.3 Step I: Explore a Corrective Factor for Widely-varied Body Sizes

CCT-measured values (LVEDV, LVMM, and MAA) of the LV varied widely among children of different ages. Therefore, CCT measurements were adjusted for body size. Factors representing body size included age, height, weight, and body surface area (BSA). The correlation coefficients of each factor for all CCT measurements were checked. The factor with the highest correlation coefficient was used in Step II to adjust the normal LV quality in children of different ages and widely-varied LV sizes.

### 2.4 Step II: Build Normal Reference Curves and Formulas of LV Quality

Using CCT-measured LV characteristics adjusted by the most significantly correlated body size factor obtained from Step I, we established the body-size-adjusted “normal range” references of all three measurements. LV quality was defined as the percentage of an individual’s measurements divided by body-size-adjusted normal values.

### 2.5 Step III: Demonstrate the Characteristics of LV Quality in DRV

**Fig. 2.**
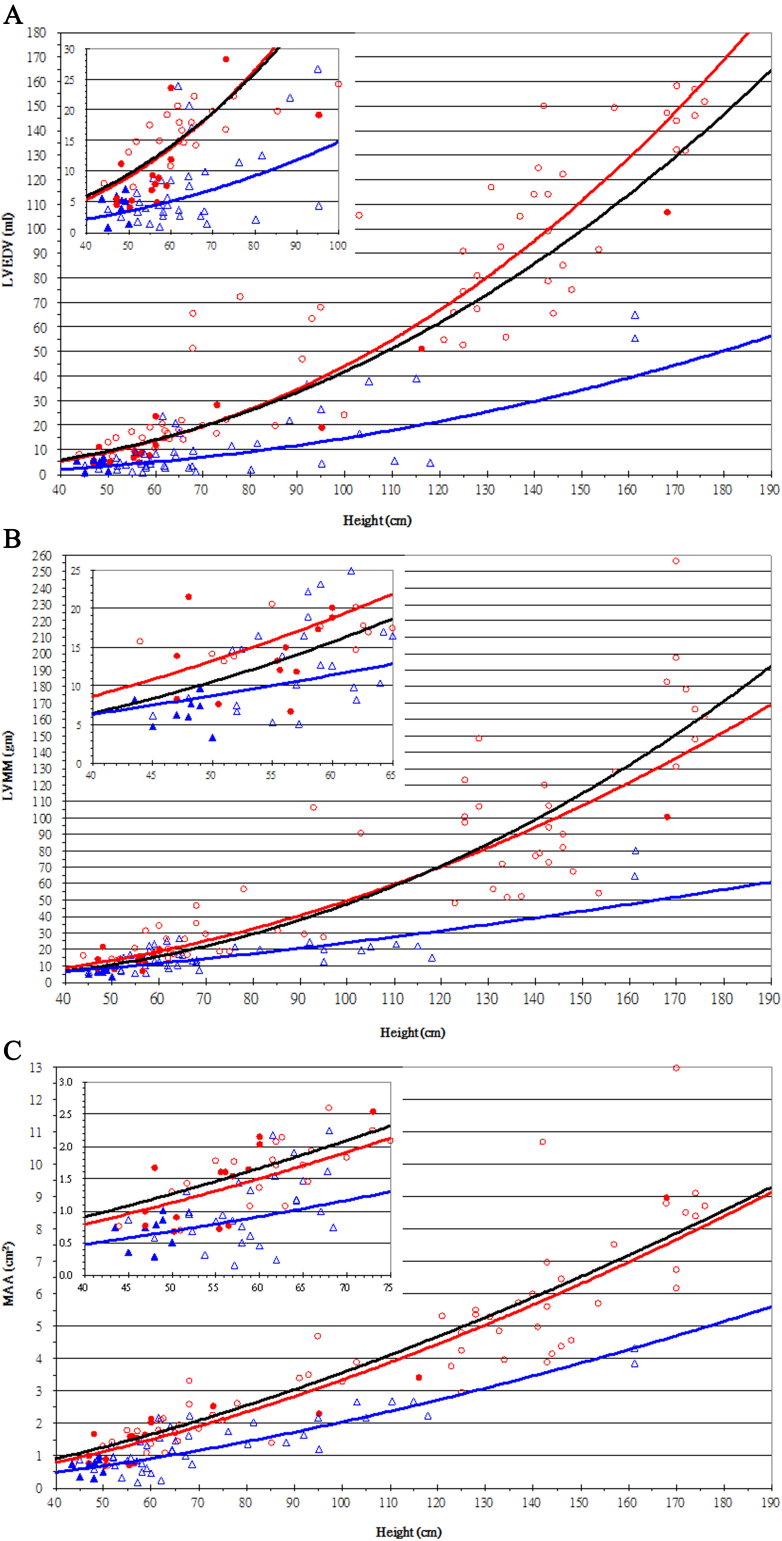
**Mean regression curves of LV measurements in different groups**. Black lines are the normal reference curves. Red lines and blue lines are mean regression curves of patients who survived after biventricular repair or single ventricle palliation, respectively. Scatter plots of LVEDV (A), LVMM (B), and MAA (C) relative to height in patients with a dominant right ventricle, who survived after their final surgical correction, against normal references (black regression lines). The patients who underwent BiVR are represented by red circles and those who underwent SVP are represented by blue triangles, with their regression estimations in red and blue lines. Solid markers denote measurements of patients before any intervention. The insets at the left upper corners of each figure show the case distribution in early childhood. LVEDV, left ventricular end-diastolic volume; LVMM, left ventricular myocardial mass; MAA, mitral annulus area.

For Step III, three major patient groups were established from the study group with DRV: the double outlet right ventricle (DORV), unbalanced atrioventricular septal defect (ubAVSD), and hypoplastic left heart syndrome (HLHS) groups. We only included patients who reached their final BiVR or SVP status. Each patient’s LV quality was calculated during every examination, and the calculated data was marked on the figures of normal reference curves made in Step II to use for comparison (Fig. 2). This served to reveal any differences and tendencies in these two interventions (BiVR or SVP) in the present clinical practice, which could be represented by their own regression estimations. Finally, to explore the lowest limit of LV quality on the first visit that could predict survival from a final BiVR, we analyzed CCT measurements from patients with DRVs who had not undergone any interventions. The ranges of LV quality were compared between patients who survived and those who expired.

### 2.6 Statistical Analysis

Descriptive statistics and Student’s *t*-test were used to compare the mean measurements in the study and control groups. A two-tailed Pearson correlation coefficient (*r*) of >0.8 was considered significant. Reference curves were plotted using general linear regression analysis according to the highest coefficient of determination ($R^{2}$) values obtained in the curve with the best fit. Statistical analyses were performed using SPSS (version 16.0, SPSS Inc., Chicago, IL, USA). *p *< 0.05 was considered statistically significant.

## 3. Results

### 3.1 Step I: Height Exhibited the Highest Correlation

We included 76 controls (age range: 27 days–20.7 years, mean 9.6 years; female: male = 25:51) with “normal” hearts (Table 1). Height exhibited the highest correlation with all measurements. The correlation coefficients of height with LVEDV, LVMM, and MAA were 0.93, 0.87, and 0.90, respectively, (*p *< 0.001) (**Supplementary Table 1**).

**Table 1.** **Participant characteristics**.

Characteristics	Healthy Controls (n = 76 pt/76 ex)	DRV	
		BiVR	SVP
		(n = 22 pt/95 ex)	(n = 8 pt/50 ex)
Mean age	9.6 yr	5.6 yr	2.1 yr
(range)	(27 d–20.7 yr)	(1 d–18.9 yr)	(1 d–28.8 yr)
Male	51	14	4
(%)	(67.1%)	(63.6%)	(50.0%)
DORV		(n = 8 pt/72 ex)	(n = 3 pt/17 ex)
HLHS			(n = 4 pt/27 ex)
HLHS*		(n = 3 pt/9 ex)	
ubAVSD		(n = 9 pt/12 ex)	(n = 1 pt/6 ex)
ubAVSD*		(n = 2 pt/2 ex)	

* = expired; BiVR, biventricular repair; DORV, double outlet right ventricle; DRV, dominant right ventricle; ex, examination; HLHS, hypoplastic left heart syndrome; pt, patient; SVP, single ventricle palliation; ubAVSD, unbalanced atrioventricular septal defect.

### 3.2 Step II: Build “Normal” Reference for LV Quality

The study group (N = 76) remained the same as that used in Step I. In Step I, height exhibited the strongest correlation with all three CCT measurements (LVEDV, LVMM, and MAA). By using “height” as an independent variable, we established the normal reference curves of the CCT-measured LV quality (black regression lines, Fig. 2) and their formulas as follows:

(1) $$\begin{array}{c} \text{𝐋𝐕𝐄𝐃𝐕}\left(\mathrm{mL}\right)=0.0022826558\times {\left(\text{𝐡𝐞𝐢𝐠𝐡𝐭}\right)}^{2.1316673352}\ldots \left[{\text{R}}^{2}=0.933\right]\\ \text{𝐋𝐕𝐌𝐌}\left(g\right)=0.0021273778\times {\left(\text{𝐡𝐞𝐢𝐠𝐡𝐭}\right)}^{2.1748578761}\ldots \left[{\text{R}}^{2}=0.868\right]\\ \text{𝐌𝐀𝐀}\left({\mathrm{cm}}^{2}\right)=0.0069124642\times {\left(\text{𝐡𝐞𝐢𝐠𝐡𝐭}\right)}^{1.3680602272\,\ldots }\ldots \left[{\text{R}}^{2}=0.858\right] \end{array}$$

### 3.3 Step III: Characteristics of LV Quality in DRV

The scatter plots of each patient’s LV quality are shown in Fig. 2. The plots show the regression estimations of LV quality in the two subgroups (BiVR vs. SVP) of patients who survived after their final surgical correction. Curves representing BiVR (in red) or SVP (in blue) differed significantly in all LV measurements.

From the regression curves, we found that the initial LVEDV of patients who received BiVR was similar to that of healthy subjects with shorter heights and of younger ages (Fig. 2A). The initial LVMM of the younger patients who received BiVR was slightly heavier than normal. However, this population had a slower increase in LV mass, which eventually became lighter than normal when the patients became taller or older (Fig. 2B). Patients who underwent BiVR always exhibited an MAA similar to that of healthy subjects, even after reaching adulthood (Fig. 2C). Those who initially received SVP had smaller LVEDV, lighter LVMM, and smaller MAA, did not “catch up” as they grew up, and always lagged below the normal range (Fig. 2). A comparison of the initial LV quality of the patients with DRV between BiVR (solid red circles in Fig. 2) and SVP (solid blue triangles in Fig. 2) survival revealed some overlapping LV measurements at similar body statuses. This representation reflects the current clinical practice. It is possible that some patients who do not have poor LV quality should still be considered for BiVR.

To explore the lowest limit of the LV quality that could survive BiVR, we included patients with a DRV who expired or survived after BiVR for further analysis (Table 2). In the DORV group, the mean of all three LV quality values were higher in the BiVR group than those in the SVP group (LVEDV, 92.9% vs. 71.0%; LVMM, 140.9% vs. 93.7%; and MAA, 86.2% vs. 63.3%); however, only MAA differed significantly between the groups (*p *< 0.05). In the HLHS group, the LV quality of patients who expired after BiVR and of those who survived SVP did not differ significantly (LVEDV, 40.7% vs. 45.6%; LVMM, 55.7% vs. 65.5%; MAA, 44.7% vs. 41.5%). In the ubAVSD group, patients who survived after BiVR exhibited a considerably better LV quality than that of those who underwent SVP (LVEDV, 63.6% vs. 13.8%; LVMM, 87.3% vs. 31.7%; MAA, 78.7% vs. 35.0%). The patients with ubAVSD who expired after BiVR exhibited significantly lower LVEDV (15.9% vs. 63.6%; *p *< 0.05) and LVMM (49.4% vs. 87.3%; *p *< 0.05) than those of patients who survived; however, the MAA did not differ significantly (58.7% vs. 78.7%) from the survivors. The two patients with ubAVSD who expired after BiVR exhibited slightly higher initial mean LV quality than the single patient with ubAVSD who survived after SVP (LVEDV: 15.9% vs. 13.8%; LVMM: 49.4% vs. 31.7%; MAA: 58.7% vs. 35.0%).

**Table 2.** **LV adequacy in patients before any intervention**.

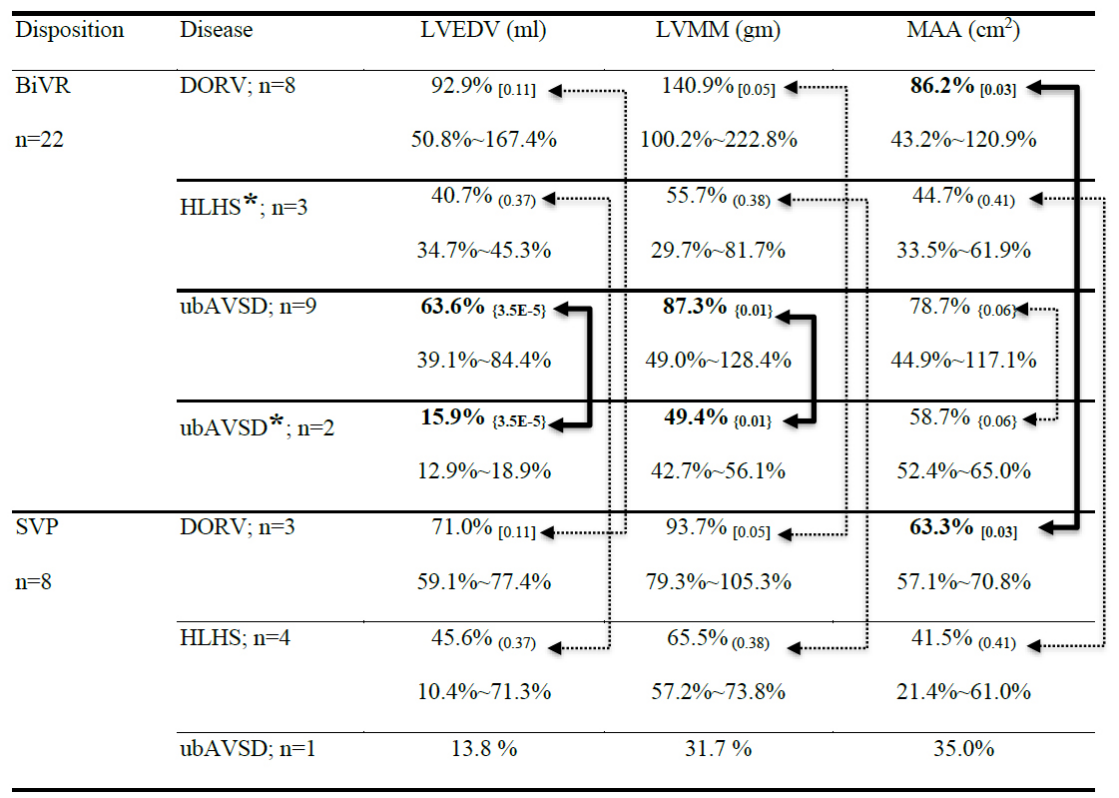

* = expired; BiVR, biventricular repair; DORV, double outlet right ventricle; HLHS, hypoplastic left heart syndrome; LVEDV, left ventricular end-diastolic volume; LVMM, left ventricular myocardial mass; MAA, mitral annulus area; SVP, single-ventricle palliation; ubAVSD, unbalanced atrioventricular septal defect; subscripted [ ] = *p* values between BiVR to SVP on DORV; subscripted ( ) = *p* values between expired BiVR to survived SVP in patients with HLHS; subscripted { } = *p* values between survived and expired patients with ubAVSD after BiVR. Bold characters and bold double arrow lines indicate pair comparisons (*p *< 0.05). Double arrow dotted lines indicate lack of statistical significance in those pair comparisons.

## 4. Discussion

Dominant right ventricle has remained challenging concerning diagnosis and surgical management despite improved outcomes in patients with the balanced form [25, 26]. From a diagnostic standpoint, DRV has been previously diagnosed primarily by ventricular size; however, unbalance can be present even if the contralateral ventricle is not particularly small [4, 27, 28]. In DRV, DORV, HLHS, and ubAVSD have very different anatomical factors that affect the feasibility of BiVR. Presently, in our hospital the decision for BiVR is made according to the clinical experience of the surgeon as well as some echocardiographic measurements and scores [3, 4, 5, 6, 7, 8, 9, 10, 11]. However, in a recently reviewed article shows these measurements or scores have only limited clinical relevance [25]. We propose multiple novel factors (mitral inflow, size and type of ventricular septal defect, and atrioventricular valve regurgitation) should be considered before opting for BiVR in patients with a DRV. We think the LV comprises three major parts: inlet, ventricle proper, and outlet. The LVMM represents the LV power that can be provided. Because the outlet of the LV in CHD can be modified using numerous modern surgical techniques, we propose that the anatomical characteristics of the size of the mitral annulus, volume of the LV cavity, and mass of the LV myocardium are the key factors for quantifying LV quality on CCT images.

Reported LV quality has mainly been assessed using echocardiography during infancy [3, 4, 5, 8, 10]. An LVEDV index >15 mL/$m^{2}$ was reported as favorable for BiVR [6]. In our study, the LVEDV index value of 15 mL/$m^{2}$ was approximately 40% of the normal reference in infancy. However, the LVEDV index varies as children grow taller (**Supplementary Fig. 1** and **Supplementary Table 1**); hence, it cannot be used after infancy. Our height-adjusted normal reference curves and formulas are highly valuable and provide a tool to evaluate LV quality for BiVR decisions both during infancy and before completion of the Fontan procedure in older children (**Supplementary Table 2**).

This study highlighted trends that have not previously been reported in patients with DRVs who have reached their final disposition of either BiVR or SVP. First, the results show how the decision between BiVR and SVP is made based on the characteristics of the LV quality. Clearly, the mean characteristics of LV quality in the surviving infants were always higher in patients who underwent BiVR than those in patients who underwent SVP. In patients with ubAVSD, sufficiently higher values of LV quality appeared to promise sustainability of the LV and survival in those who underwent BiVR. Alternatively, SVP could be selected for patients to ensure survival. However, our study revealed an overlap in the range of the LV quality. In patients with HLHS, LV quality measurements did not differ significantly between the surviving patients who underwent SVP and expired patients who underwent BiVR. Therefore, our study showed that SVP was a safe procedure for improving patient survival.

The development of LV quality after BiVR or SVP differed in our study. Overall, the LV quality was poorer, and the LV grew slower in the SVP than in the BiVR subgroups. Such differences in the LV development after BiVR or SVP imply that reducing blood flow can impair LV growth in the long term. We found, compatible with other studies, the ability of the left-sided heart structures in DRV patients to have catch-up growth after BiVR [29, 30, 31, 32, 33]. However, the three biomarkers of LV quality showed differences in development in the BiVR subgroup. The MAA neared the normal reference, LVMM became initial thicker but longterm change to lighter or thinner, and LVEDV increased or the LV was more dilated than the normal references. Reduction in LV mass and dilation of the LV chamber may be early signs of LV failure, which may be addressed by further long-term follow up.

The patterns of LV quality differed before any intervention in the three major subgroups. In DORV, the pathological right ventricle is often abnormally dilated, making the LV look relatively small. Our data show that the LV quality was actually not as poor as expected [27, 28]. Some individuals even exhibited better LV quality than that in healthy individuals (i.e., >100%). However, in HLHS, an abnormal LV is inherent, and all LV measurements are poor. Unfortunately, all patients with HLHS in our study who underwent BiVR expired. These results show that the LV quality in patients with HLHS should be considerably higher than that of patients in our present study when considering BiVR in the future.

The patients with ubAVSD represented the only study group that had both surviving and deceased patients after BiVR, as well as one surviving patient after SVP. Patients who survived after BiVR had significantly higher LVEDV and LVMM values than those in patients who did not survive. The lowest limits of the LVEDV, LVMM, and MAA ranges were 39.1%, 49.0%, and 44.9%, respectively, which are all data from the identical one of the nine surviving patients. So, we propose at least 44.9% of all three parameters in one patient is the lowest limited to promise BiVR in DRV. A previous reported LVEDV index of >15 mL/$m^{2}$ which was considered more favorable for BiVR is approximately 40% of our normal reference data in infancy [6]. Furthermore, for more convenient clinical application, we propose a minimum criterion of approximately 45% as the cutoff point in all three CCT measurements. This means that the values in all three LV CCT measurements (LVEDV, LVMM, MAA) should be higher than approximately 45% of the normal references for improving BiVR survival; we define this as LV adequacy. This value was verified in both surviving and deceased patients. The results showed a sensitivity, a negative predictive value, and an accuracy of 94%, 88%, and 77%, respectively. We believe that having adequate LV quality and other favorable conditions is essential for a successful BiVR that promises patient survival.

Our study had several limitations. Radiation exposure is an inherent disadvantage of CCT; however, exposure has decreased with newer technology. Other factors that are crucial for BiVR were not addressed in this study, including mitral secondary inflow assessment, size and type of ventricular septal defect, atrioventricular valve regurgitation, and degree of outflow tract obstruction. The sample size was small, and follow-up studies are warranted to obtain more objective data for verification.

## 5. Conclusions

In conclusion, LV quality measured using 3D CCT could be used to guide and monitor patients with DRV before and after BiVR. We propose that the threshold of all three values of LV quality (LVEDV, LVMM, and MAA) should be at least 45% or greater to achieve better outcomes in patients with DRV in whom BiVR is being considered.

## Data Availability

The datasets generated and/or analyzed during the current study are not publicly available due to privacy of patients but are available from the corresponding author on reasonable request.

## Abbreviations

BiVR, Biventricular repair; BSA, body surface area; CCT, cardiac computed tomography; CHD, congenital heart disease; DORV, double outlet right ventricle; DRV, dominant right ventricle; HLHS, hypoplastic left heart syndrome; LV, left ventricle; LVEDV, LV end-diastole volume; LVMM, LV myocardium mass; MAA, mitral annulus area; SVP, single ventricle palliation; ubAVSD, unbalanced atrioventricular septal defect.

## Supplementary Material

Supplementary material associated with this article can be found, in the online version, at https://doi.org/10.31083/j.rcm2403092.
