# Supplementary material for: Determining Biventricular Repair Feasibility in Children with Dominant Right Ventricle Using Left Ventricular Quality Measured on Cardiac Computed Tomography
Source: Rev Cardiovasc Med. 2023 Mar 16;24(3):92. doi: 10.31083/j.rcm2403092 (PMC11263996; doi:10.31083/j.rcm2403092)
Supplement: Supplementary file 1 [file 2153-8174-24-3-092-s1.docx]

**SUPPLEMENTARY MATERIAL**

**Supplementary** **Figure 1**.

| 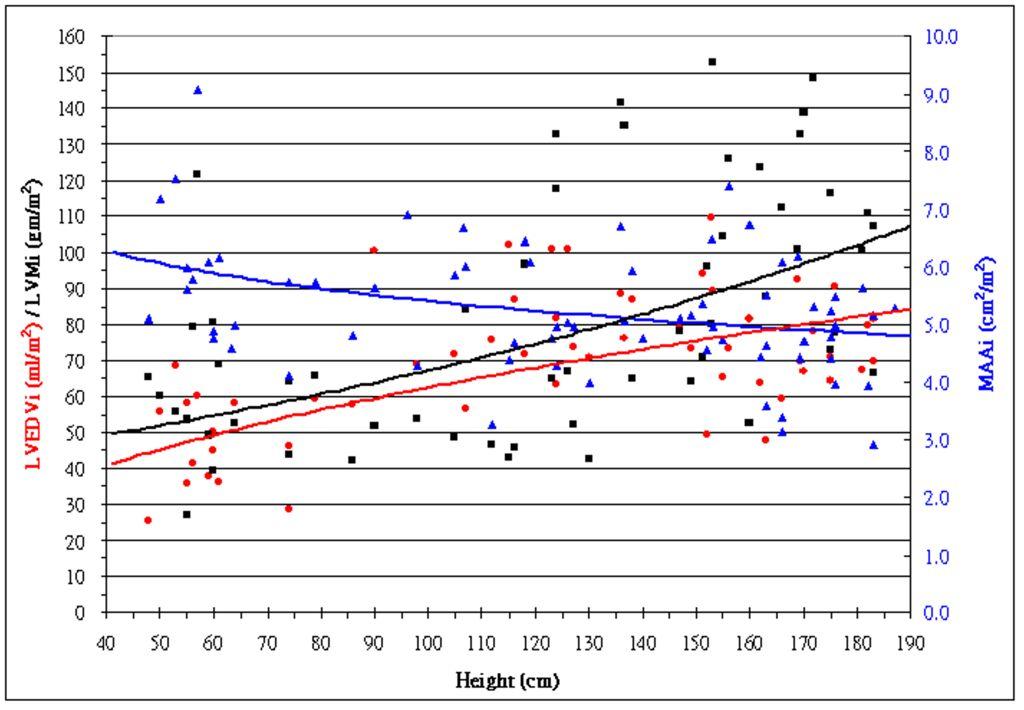 |
| --- |
| **Figure 1.**  Scatter plot distribution between body surface area (BSA)-adjusted indices of LV quality to height. We checked the relationship between BSA indices of LV quality and height by using a scatter plot distribution. This scatter plot indicated that the height of the children expressed a slightly positive correlation with the LVEDV index and LVMM index but a negative correlation with the MAA index. Therefore, setting a threshold on only one value among these indices of LV quality to differentiate between normal or abnormal LV quality is not reasonable. LVEDVi = left ventricular end-diastolic volume index (red circle and red regression line; LVEDV/BSA; mL/m^2^); LVMi = left ventricular myocardial mass index (black square and black regression line; LVM/BSA; g/m^2^); MAAi = mitral annulus area index (blue triangle and blue regression line; MAA/BSA; cm^2^/m^2^). |

**Supplementary** **Table 1**.

Pearson correlation coefficients (*r*) and *p* values (*p*) between LV characteristics relative to factors of body size

| Factors |  | LVEDV | |  | LVMM | |  | MAA | |
| --- | --- | --- | --- | --- | --- | --- | --- | --- | --- |
|  |  | *r* | *p* |  | *r* | *p* |  | *r* | *p* |
| Age |  | .87 | 3.2E-20 |  | .85 | 2.7E-18 |  | .86 | 1.3E-23 |
| **Height** |  | **.93** | **1.2E-25** |  | **.87** | **1.6E-18** |  | **.90** | **1.8E-25** |
| Weight |  | .90 | 1.4E-22 |  | .86 | 2.1E-18 |  | .89 | 3.7E-26 |
| BSA |  | .64 | 2.7E-8 |  | .63 | 5.4E-8 |  | .70 | 1.4E-12 |

The most significantly correlated factor of the body size to the LV character is represented by **bold** letters. BSA = body surface area; LV = left ventricle; LVEDV = left ventricular end-diastolic volume (ml); LVMM = left ventricular myocardial mass (g); MAA = mitral annulus area (cm^2^).

We recently applied our study result to one patient with a favorable outcome (Supplementary Table 2). This HLHS patient was initially planned to receive an SVP 3 years ago. After preparatory surgeries, she had reached a hemi-Fontan circulation. However, the quality of her left ventricular characters were acceptable during the last follow-up cardiac CT after the preliminary result of this study was obtained. We retrospectively calculated the quality of her left ventricular characters in a previously performed cardiac CT. The result provided us with the confidence to proceed with biventricular circulation. Biventricular conversion was performed on this patient; a smooth postoperative course was observed, and she was discharged from hospital with satisfactory activity.

| **Supplementary** **Table 2**.   \| Age \| LVEDV \| LVMM \| MAA \| \| --- \| --- \| --- \| --- \| \| 15 ds \| 45.1 % \| 63.0 % \| 46.0 % \| \| 18 ds \| Norwood I+ RV-PA shunt+ divided PDA+ TV valvuloplasty \| \| \| \| 2.3 ms \| 40.7 % \| 83.2 % \| 60.2 % \| \| 4.0 ms \| 67.2 % \| 89.9 % \| 67.1 % \| \| 4.2 ms \| Bilateral Glenn shunts+ atrial septectomy+ RV-PA shunt division  + TV annuloplasty \| \| \| \| 6.1 ms \| 64.6 % \| 65.7 % \| 63.9 % \| \| 3.1 ys \| 67.2 % \| 51.6 % \| 69.8 % \| \| 3.3 ys \| Take down Glenn shunts+ patches repaired ASD and VSD  + aortoplasty+ PA augmentation \| \| \| |
| --- | --- | --- | --- | --- | --- | --- | --- | --- | --- | --- | --- | --- | --- | --- | --- | --- | --- | --- | --- | --- | --- | --- | --- | --- | --- | --- | --- | --- | --- | --- | --- | --- | --- | --- | --- | --- |
| Biventricular conversion on a 3.3 years old patient who had been planned to a Fontan circulation in her infancy. ASD= atrial septal defect; CT= computed tomography; ds= days; LVEDV = left ventricle end-diastole volume; LVMM = left ventricle myocardial mass; MAA = mitral annulus area; ms = months; PA = pulmonary artery; PDA = patent ductus arteriosus; RV = right ventricle; TV = tricuspid valve; VSD = ventricular septal defect; ys = years. |
